# Public Health Actions to Control Measles Among Afghan Evacuees During Operation Allies Welcome — United States, September–November 2021

**DOI:** 10.15585/mmwr.mm7117a2

**Published:** 2022-04-29

**Authors:** Nina B. Masters, Adria D. Mathis, Jessica Leung, Kelley Raines, Nakia S. Clemmons, Kathryn Miele, S. Arunmozhi Balajee, Tatiana M. Lanzieri, Mona Marin, Deborah L. Christensen, Kevin R. Clarke, Miguel A. Cruz, Kathleen Gallagher, Shannon Gearhart, Alida M. Gertz, Onalee Grady-Erickson, Caroline A. Habrun, Gimin Kim, Michael H. Kinzer, Shanna Miko, M. Steven Oberste, Julia K. Petras, Emily G. Pieracci, Ian W. Pray, Hannah G. Rosenblum, James M. Ross, Erin E. Rothney, Hannah E. Segaloff, Leah V. Shepersky, Kimberly A. Skrobarcek, Anna M. Stadelman, Kelsey M. Sumner, Michelle A. Waltenburg, Michelle Weinberg, Mary Claire Worrell, Noelle E. Bessette, Lilian R. Peake, Marshall P. Vogt, Meredith Robinson, Ryan P. Westergaard, Richard H. Griesser, Joseph P. Icenogle, Stephen N. Crooke, Bettina Bankamp, Scott E. Stanley, Paul A. Friedrichs, Larry D. Fletcher, Iván A. Zapata, Herbert O. Wolfe, Pritesh H. Gandhi, Julia Y. Charles, Clive M. Brown, Martin S. Cetron, Nicki Pesik, Nancy W. Knight, Francisco Alvarado-Ramy, Michael Bell, Leisel E. Talley, Lisa D. Rotz, Paul A. Rota, David E. Sugerman, Paul A. Gastañaduy, Operation Allies Welcome Response Group, Operation Allies Welcome Response Group, Indu B. Ahluwalia, Olubunmi A. Akinkugbe, Aaron Aranas, Melissa Arons, Christine Atherstone, Valerie Bampoe, Patricia Bessler, Leticia Bligh, Kimberly Bonner, Virginia B. Bowen, Kendra Broadwater, Gary W. Brunette, Joan M. Brunkard, Dru A. Burns, Molly Cantrell, Bryan E. Christensen, James R. Cope, Janine Cory, Nathan E. Crawford, David Daigle, Scott M. Daly, Peter Dejonge, Mushtaq Dualeh, Kevin H. Dunn, Rachel B. Eidex, Kai Elgethun, Geroncio Fajardo, Maureen Fonseca-Ford, Katherine Franc, Joanna Gaines, Nisha George, James Goodson, Caitlin Green, Aaron J. Grober, Kibrten Hailu, Duane R. Hammond, Brian H. Harcourt, Alexandra Hess, Elisabeth Hesse, Deborah V.L. Hirst, Jennifer Hornsby-Myers, Ben Humrighouse, Maliha Ishaq, Kenta Ishii, Allison James, Bina Jayapaul-Philip, Emily S. Jentes, Latoya Johnson, Marie Johnston, Christine D. Jolley, Akadia Kacha-Ochana, Harpriya Kaur, Megan Keaveney, Hilary C. Kelly, Vikram Krishnasamy, Gayathri S. Kumar, Ma’isah Larkin, Mary Layde, Ryan F. LeBouf, Deborah Lee, Rosa C. Lira, Raymond Lopez, Matthew J. Lozier, Alicia Macler, Hugh Mainzer, Deborah Malden, Jason Malenfant, Nina Marano, Zachary Marsh, Oren Mayer, Robert McDonald, Neha Mehta, Akshara N. Menon, Erika Meyer, Stephen T. Miles, Faisal Minhaj, Sara Mirza, Kyle M. Moller, Sapna Bamrah Morris, Dylan T. Neu, Lisa P. Oakley, Denisse Vega Ocasio, Taylor Osborne, Alan C. Ou, Megan Peck, Marissa Person, Drew Posey, Alexis Pullia, Chaolong Qi, Amanda J. Raziano, Malia Richmond-Crum, Shahrokh Roohi, John M. Saindon, Samira Sami, Liliana Sanchez-Gonzalez, Ryan Schweitzer, Amee M. Schwitters, Mays Shamout, Caitlin E. Shockey, Talya Shragai, Kimberly B. Singler, Erica J. Sison, Dallas Smith, Matthew Smith, Neha J. Sood, Brittany J. Sunshine, Alma Trujillo, Snigdha Vallabhaneni, Alexandra Wickson, Jonathan S. Yoder, Laura R. Zambuto, Tara Cozzarelli, Morrisa Rice, Madia Ricks, John S. Birchfield, Atmaram Nambiar, Avery Avrakatos, Timothy P. Ballard, Ethan Dennis, Kelly Gambino-Shirley, Amanda E. Huston, Melissa G. Jennings, David M. Oldham, Michael J. Rabener, Matthew N. Fandre, Raymond J. Jablonka, Amber Love, Olivia L. Peduzzi, Kirsten Snow, Joy A. Greer, Chase A. Hughes, Matthew A. Humphreys, Adrian B. Korduba, Kari A. Neamand-Cheney, Nikki L. Pritchard, Alisha M. Smith, Jessica L. Whelpley, Sulola Adekoya, Victoria Alexander, Meredith Davis, Jonathan Falk, Katie Kurkjian, Elizabeth McCarty, James Moss, Angela Myrick-West, Chandni Patel, Rhonda Pruitt, Dawn Saady, Denise Sockwell, Amanda Touma, Stephanie Wheawill, Diane Woolard, Andrea Young, Latoya Griffin-Thomas, Sean Kelly, Jamie McLeod, Matthew C. Lambert, Tonya L. Danz, Timothy Davis, Kyley Guenther, Erika Hanson

**Affiliations:** ^1^CDC Operation Allies Welcome Response Team; ^2^Epidemic Intelligence Service, CDC; ^3^Wisconsin Department of Health Services; ^4^Career Epidemiology Field Officer Training Program, CDC; ^5^New Mexico Department of Health; ^6^New Jersey Department of Health; ^7^Virginia Department of Health; ^8^Wisconsin State Laboratory of Hygiene, Madison, Wisconsin; ^9^U.S. Department of Homeland Security, Washington, DC; ^10^U.S. Department of Defense, Washington, DC.; CDC; CDC; CDC; CDC; CDC; CDC; CDC; CDC; CDC; CDC; CDC; CDC; CDC; CDC; CDC; CDC; CDC; CDC; CDC; CDC; CDC; CDC; CDC; CDC; CDC; CDC; CDC; CDC; CDC; CDC; CDC; CDC; CDC; CDC; CDC; CDC; CDC; CDC; CDC; CDC; CDC; CDC; CDC; CDC; CDC; CDC; CDC; CDC; CDC; CDC; CDC; CDC; CDC; CDC; CDC; CDC; CDC; CDC; CDC; CDC; CDC; CDC; CDC; CDC; CDC; CDC; CDC; CDC; CDC; CDC; CDC; CDC; CDC; CDC; CDC; CDC; CDC; CDC; CDC; CDC; CDC; CDC; CDC; CDC; CDC; CDC; CDC; CDC; CDC; CDC; CDC; CDC; CDC; CDC; CDC; CDC; CDC; CDC; CDC; CDC; CDC; CDC; CDC; CDC; CDC; CDC; CDC; CDC; CDC; CDC; CDC; Defense Health Agency; Health Resources and Services Administration; Health Resources and Services Administration; North American Aerospace Defense Command and U.S. Northern Command; Pennsylvania Department of Health; U.S. Agency for International Development; U.S. Air Force; U.S. Air Force; U.S. Air Force; U.S. Air Force; U.S. Air Force; U.S. Air Force; U.S. Air Force; U.S. Army; U.S. Army; U.S. Marine Corps; U.S. Marine Corps; U.S. Marine Corps; U.S. Navy; U.S. Navy; U.S. Navy; U.S. Navy; U.S. Navy; U.S. Navy; U.S. Navy; U.S. Navy; Virginia Department of Health; Virginia Department of Health; Virginia Department of Health; Virginia Department of Health; Virginia Department of Health; Virginia Department of Health; Virginia Department of Health; Virginia Department of Health; Virginia Department of Health; Virginia Department of Health; Virginia Department of Health; Virginia Department of Health; Virginia Department of Health; Virginia Department of Health; Virginia Department of Health; Virginia Department of Health; Virginia Division of Consolidated Laboratory Services; Virginia Division of Consolidated Laboratory Services; Virginia Division of Consolidated Laboratory Services; Wisconsin Department of Health Services; Wisconsin State Laboratory of Hygiene; Wisconsin State Laboratory of Hygiene; Wisconsin State Laboratory of Hygiene; Wisconsin State Laboratory of Hygiene.

On August 29, 2021, the United States government oversaw the emergent establishment of Operation Allies Welcome (OAW), led by the U.S. Department of Homeland Security (DHS) and implemented by the U.S. Department of Defense (DoD) and U.S. Department of State (DoS), to safely resettle U.S. citizens and Afghan nationals from Afghanistan to the United States. Evacuees were temporarily housed at several overseas locations in Europe and Asia* before being transported via military and charter flights through two U.S. international airports, and onward to eight U.S. military bases,^†^ with hotel A used for isolation and quarantine of persons with or exposed to certain infectious diseases.^§^ On August 30, CDC issued an Epi-X notice encouraging public health officials to maintain vigilance for measles among Afghan evacuees because of an ongoing measles outbreak in Afghanistan (25,988 clinical cases reported nationwide during January–November 2021) (*1*) and low routine measles vaccination coverage (66% and 43% for the first and second doses, respectively, in 2020) (*2*).

On September 4, CDC was notified of a suspected measles case in an Afghan evacuee at Fort McCoy, Wisconsin. In partnership with state and local health departments, CDC provided technical guidance to DHS, DoD, and DoS to increase measles surveillance, conduct case investigations and contact tracing, and implement a mass measles vaccination campaign at overseas and domestic military bases and hotel A. Among 72,299 evacuees, 47 (0.065%) confirmed measles cases were reported from August 29 (the beginning of OAW) through November 26.^¶^ Vaccination efforts across domestic and overseas locations that achieved an estimated 96% coverage with measles, mumps, and rubella (MMR) vaccine in this evacuee population were critical in limiting measles importations into the United States and preventing subsequent spread at military bases and into U.S. communities.

## Investigation and Results

Measles was first diagnosed in an evacuee aged 17 years who began experiencing prodromal symptoms on September 1 in Germany while awaiting transport to the United States (disclosed on later interview). The patient traveled on military flights from Ramstein Air Base, Germany to Washington Dulles International Airport (IAD) on September 3, and from IAD to Fort McCoy, Wisconsin on September 4. A few hours after completing the intake process, the patient sought care at the on-site acute care clinic with a fever of 107.6°F (42°C) and a maculopapular rash (Box). The patient was transferred to a local emergency department where specimens were collected for measles testing, which was performed by the Wisconsin State Laboratory of Hygiene. Upon return to Fort McCoy on September 5, the patient was isolated at an on-site facility. Measles was confirmed by real-time reverse transcription–polymerase chain reaction (RT-PCR) on September 5; molecular characterization yielded genotype B3, consistent with genotypes recently identified in countries neighboring Afghanistan.**

BOX **Time line of events associated with measles cases detected among Afghan evacuees during Operation Allies Welcome — United States and Afghanistan, August 17–October 15, 2021**

**August 17**

Flights with Afghan evacuees began arriving in the United States at IAD.

**August 28**

Flights with Afghan evacuees began arriving at PHL.

**August 24–September 24**

MMR and varicella vaccination efforts began, transitioning to rapid scale-up of vaccination on September 6 after the first measles case was identified and accelerated mass vaccination campaigns after the September 14 directive. Mass vaccination campaigns continued until September 24 across U.S. military bases and hotel A.

**August 30**

Epi-X notice issued to encourage U.S. health departments and clinicians to maintain vigilance for measles and polio among Afghan evacuees.

**September 2**

Rash onset in earliest measles case (patient aged 9 months) at hotel A, Virginia (laboratory confirmed September 9).

**September 4**

Rash onset in first identified measles case (patient aged 17 years) at Fort McCoy, Wisconsin (laboratory confirmed September 5).

**September 10**

International flights carrying OAW evacuees to the United States temporarily halted.

**September 14**

CDC directive issued to pause international evacuation flights from overseas locations to the United States and initiate mass MMR and varicella vaccination campaigns and quarantine for 21 days following receipt of MMR vaccine; total of six measles cases confirmed by this date.

**September 17**

Executive Order issued adding measles to the list of federally quarantinable communicable diseases.

**September 20**

CDC issued Health Alert Network Update: Guidance for Clinicians Caring for Individuals Recently Evacuated from Afghanistan;* total of 16 measles cases confirmed by this date.

**October 5**

Flights resumed to United States (PHL).

**October 15**

Rash onset in last measles case at Marine Corps Base Quantico, Virginia; total of 47 measles cases identified.

**Abbreviations:** Epi-X = Epidemic Information Exchange; IAD = Dulles International Airport; MMR = measles, mumps, and rubella; OAW = Operation Allies Welcome; PHL = Philadelphia International Airport.

* https://emergency.cdc.gov/han/2021/han00452.asp

A confirmed measles case was defined as an acute febrile rash illness and either detection of measles virus RNA using real-time RT-PCR or measles-specific immunoglobulin M antibody by enzyme immunoassays, or direct epidemiologic linkage to a laboratory-confirmed case (*3*). By September 14, five additional measles cases had been confirmed: four in patients at hotel A and one in a patient at Fort Pickett, Virginia. To identify additional cases, active case finding and tracing of exposed contacts were conducted by DoD and DHS public health surveillance and medical staff members and contractors at domestic military bases, international airports, and hotel A. This activity was reviewed by CDC and was conducted consistent with applicable federal law and CDC policy.^††^

A total of 47 confirmed measles cases were reported among Afghan evacuees at six domestic sites in four jurisdictions (22 cases in Virginia, 22 in Wisconsin, two in New Mexico, and one in New Jersey). Rash onset dates ranged from September 2 to October 15.^§§^ The median age of patients was 1 year (range = 0–26 years); 16 (34%) patients were aged <12 months, 17 (36%) were aged 1–4 years, 11 (23%) were aged 5–19 years, and three (6%) were aged 20–29 years; 55% were male. All 47 patients were unvaccinated or had unknown vaccination status upon arrival in the United States. Overall, 46 (98%) cases were laboratory-confirmed. Genotyping performed on 43 real-time RT-PCR–positive specimens identified genotype B3 in all. The crude attack rate was 0.065%. Domestic sites also reported 57 varicella cases, 14 mumps cases, and one rubella case.^¶¶^

## Public Health Response

After DoD began larger-scale emergency evacuations, administration of routine predeparture vaccinations in Afghanistan was not operationally feasible because of the urgency and scope of the evacuations, and efforts shifted to providing MMR vaccine to evacuees soon after arrival in the United States. Following the detection of measles cases, rapid scale-up of DoD-led vaccination efforts began across domestic military bases housing evacuees on September 6, and evacuation flights from overseas locations to the United States were temporarily halted on September 10. CDC issued a directive on September 14 recommending urgent implementation of measures to limit measles spread; this directive included a pause on evacuation flights from overseas locations and the acceleration of mass MMR and varicella vaccination for all eligible evacuees aged ≥6 months and ≥12 months, respectively, who did not have contraindications (*4*,*5*), at both OAW overseas and domestic locations. Because of the lack of documentation of previous vaccination or disease history of evacuees, more targeted vaccination of susceptible persons was not possible. In addition, all evacuees were presumed to have been exposed to measles and thus were recommended to remain in quarantine (i.e., on bases and in overseas locations) for 21 days following receipt of MMR vaccine (*3*). Efforts were also made to provide immunoglobulin to persons ineligible for MMR vaccine (infants aged <6 months and seronegative pregnant women) at domestic sites.

By September 24, an estimated 91% of eligible evacuees at the domestic military bases had been vaccinated with MMR vaccine, increasing to 96% by November 25. No measles cases were reported in military personnel, volunteers and staff members supporting the OAW response, or in the community (areas surrounding the military bases or hotel A); no measles-related deaths occurred. OAW international flights resumed on October 5, with no measles cases identified among evacuees arriving after the pause.

An additional tool for measles control became available on September 17 with the addition of measles to the list of federally quarantinable diseases via Executive Order 14047.*** This policy change enabled the use of federal regulatory authority to control measles transmission by allowing the issuance of federal public health orders, if necessary. CDC developed operating procedures and policies to determine situations for which issuing a federal order for isolation of patients and quarantine of exposed contacts might be necessary for the protection of public health. As a result of the high measles vaccination coverage achieved through this response and the adherence with voluntary isolation and quarantine recommendations, no federal public health orders were issued.

## Discussion

Measles is an extremely contagious viral illness, with one infectious patient capable of infecting an average of 12–18 persons in a fully susceptible population (*6*). Measles-containing vaccines are highly effective, with 2 doses conferring approximately 97% protection (*4*). Measles elimination^†††^ has been maintained in the United States since 2000; however, measles cases reached a 25-year high in 2019, with 1,274 cases reported across 32 U.S. jurisdictions; 85% of these cases occurred among pockets of undervaccinated persons, where the virus spread following international importations (*7*). Reduced global travel, physical distancing, and other COVID-19 pandemic-related mitigation measures likely contributed to only 15 measles cases being reported in the United States from 2020 to 2021 before the start of OAW (*8*). However, the lessons of the 2019 U.S. outbreaks highlight the need for vigilance as well as the importance of prompt interventions to control measles (*8*). In addition, global standards for humanitarian crises^§§§^ recommend conducting a mass measles vaccination campaign when estimated measles coverage is ≤90% or is not known. The low measles vaccination coverage among evacuees, coupled with the high potential for multiple importations, increased risk for transmission in congregate settings, and possible spread into U.S. communities during the OAW response, demanded immediate public health action requiring a whole-of-government approach.

All identified cases occurred among evacuees who arrived during August 17–September 10 before international flights from overseas locations were temporarily halted to permit mass vaccination of all evacuees. The absence of additional cases in evacuees who arrived after flights resumed is evidence of the success of this strategy in preventing new introductions of measles into the United States. This response also highlights the effectiveness of the mass vaccination campaign in minimizing further transmission on military bases (attack rates for measles outbreaks among refugee populations in congregate settings have ranged from 0.9% to 25.5%) (*9*,*10*), as well as preventing transmission into communities with health care systems already strained by the COVID-19 pandemic. Efforts continued to 1) ensure high vaccination coverage among the remainder of incoming evacuees, 2) identify and isolate ill persons among evacuees on military bases, and 3) perform contact tracing to identify and quarantine exposed persons.

The findings in this report are subject to at least two limitations. First, cases might have been missed because of clinical misdiagnoses, limited available staff members to conduct timely and regular wellness checks, and failure of ill persons to seek care at acute care clinics. Second, contact tracing and ascertainment of exposures were difficult because of evacuees mixing at overseas locations, on international and domestic flights, at receiving airports, and at military bases, creating challenges for adequate monitoring among exposed persons.

Rapid implementation of a high-coverage mass measles vaccination campaign by DoD with a 21-day quarantine after receipt of MMR vaccine reduced measles importations and prevented substantial potential spread of measles on military bases and into U.S. communities, and the morbidity and mortality associated with such outbreaks. The robust MMR and varicella vaccination campaign also likely limited the number of varicella, mumps, and rubella cases identified across military bases.

**Summary**

**What is already known about this topic?**

Low measles immunization coverage and an ongoing measles outbreak in Afghanistan led to U.S. measles importations among Afghan evacuees who were resettled as part of Operation Allies Welcome.

**What is added by this report?**

Forty-seven measles cases were reported among 72,299 Afghan evacuees (attack rate = 0.065%) in U.S. military bases and a contracted hotel. A coordinated response and a high-coverage mass vaccination campaign led to rapid containment.

**What are the implications for public health practice?**

Mass vaccination of an undervaccinated evacuee population can limit measles importations, control measles spread, and prevent transmission into U.S. communities.

## Footnotes

* Measles was identified in evacuees who transited through Bahrain, Bulgaria, Germany, Italy, Kuwait, Qatar, and Spain.

† Camp Atterbury, Indiana; Fort Bliss, Texas; Fort Lee, Virginia; Fort Pickett, Virginia; Fort McCoy, Wisconsin; Holloman Air Force Base, New Mexico; Joint Base McGuire-Dix-Lakehurst, New Jersey; and Marine Corps Base Quantico, Virginia.

§ Hotel A is located in Virginia and was closed to the public and used only for quarantine and isolation during OAW.

¶ November 26 is 42 days, or two maximum incubation periods, after rash onset in the last case (October 15); a measles outbreak is considered to have ended if there are no additional outbreak-related cases for two or more maximum incubation periods after rash onset in the last case under continued surveillance.

**
https://www.who.int/teams/immunization-vaccines-and-biologicals/immunization-analysis-and-insights/surveillance/monitoring/provisional-monthly-measles-and-rubella-data

†† 45 C.F.R. part 46, 21 C.F.R. part 56; 42 U.S.C. Sect. 241(d); 5 U.S.C. Sect. 552a; 44 U.S.C. Sect. 3501 et seq.

§§ The patient with rash onset on September 2 was identified at hotel A in Virginia, and measles was laboratory confirmed on September 9 after identification and laboratory-confirmation of the first case at Fort McCoy, Wisconsin.

¶¶
https://ndc.services.cdc.gov/

***
https://www.whitehouse.gov/briefing-room/presidential-actions/2021/09/17/executive-order-on-adding-measles-to-the-list-of-quarantinable-communicable-diseases/

††† The absence of endemic measles virus transmission or a chain of measles transmission that persists for 1 year or more.

§§§
www.spherestandards.org/handbook
